# Bithalamic Infarction (Artery of Percheron Occlusion) after Anterior Cervical Discectomy and Fusion

**DOI:** 10.1155/2019/9438089

**Published:** 2019-03-18

**Authors:** Hatem B. Afana, Nidal M. M. Abuhadrous, Alaa Eldin Elsharkawy

**Affiliations:** ^1^Orthopaedic Department, European Gaza Hospital, Gaza Strip, State of Palestine; ^2^Neurosurgery Department, European Gaza Hospital, Gaza Strip, State of Palestine; ^3^Neurosurgery Department, Bonifatius Hospital, Lingen, Germany; ^4^Neurosurgery Teaching Program, University of Traditional Medicine, Yerevan, Armenia

## Abstract

Bithalamic infarction resulting from occlusion of the artery of Percheron after cervical spine surgery is a rare pathological entity. Diagnosis and early detection are challenging. Prompt management may help to improve the outcome. We present a case of a 39-year-old male patient, smoker, diagnosed with multiple cervical disc herniations, who underwent Anterior Cervical Discectomy and Fusion (ACDF) for C3-C4, C4-C5, and C5-C6. During the 2-hour and 50-minute surgery, the patient was lying supine with his neck hyperextended. The intraoperative procedure was uneventful. During surgery, blood pressure ranged around 110 mmHg∖50 mmHg. At the end of surgery, the patient's recovery from general anesthesia was normal with no delaying or complication; on next the day, patient developed a sudden loss of consciousness. Urgent brain computed tomography (CT) was normal; two days later, follow-up CT and CT Angiography (CTA) revealed bilateral thalamic infarction with right vertebral artery occlusion from its origin. Intraoperative surgical manipulation, hypotensive anesthesia, and prolonged neck hyperextension might have contributed to stroke in this patient. ACDF carries a potential risk for posterior circulation stroke. Artery of Percheron infarction should be considered in the differential diagnosis of patients developing a sudden loss of consciousness after ACDF. Vertebral artery thrombosis should be taken into account as an important possible cause of embolism.

## 1. Introduction

Anterior cervical discectomy with fusion (ACDF) is an established intervention for cervical degenerative disease [1]. ACDF is a relatively safe procedure with a very low morbidity and mortality rate [2]. However, this procedure may be associated with serious postoperative complications like strokes. Several case reports have described patients suffering strokes following ACDF [3–5]. Published data showed that patients with carotid artery stenosis have a greater incidence of postoperative stroke [5].

A postoperative stroke of the posterior circulation in a previously asymptomatic patient is a rare neurological event [6]. Thalamic infarction is relatively uncommon, it accounts for about 11% of all vertebrobasilar infarcts [7].

Bithalamic infarction after ACDF is a very rare presentation of stroke. A combination of predisposing factors and anatomic variations may be responsible for this type of stroke [7].

One of the vascular variations of the brain where a cerebral blood vessel supplies structures on both sides is the artery of Percheron. The artery of Percheron (AOP) is a rare anatomic variation in the posterior circulation of the brain in which a single arterial trunk arises from the first part of the posterior cerebral artery (PCA) and supplies the medial region of both thalami. The occlusion of the artery of Percheron results in bilateral infarctions in the middle aspects of thalami and brainstem [8, 9]. We report a case of bithalamic infarction due to embolic occlusion of the artery of Percheron as a consequence of vertebral artery (VA) thrombosis.

## 2. Case Presentation

**Figure 1:**
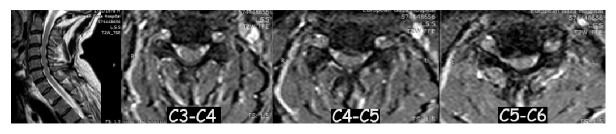
Preoperative cervical magnetic resonance image (MRI) demonstrated multilevel cervical disc with myelopathy C3-C4, C4-C5, C5-C6.

A 39-year-old male patient, smoker, presented in the outpatient clinic with chronic neck pain radiating to the right upper limb. The patient had no history of chronic disease and no previous surgeries. Cervical magnetic resonance image (MRI) demonstrated multilevel cervical disc herniation (C3-C4, C4-C5, C5-C6) with T2 high signal intensity changes (Figure 1). The patient examination was normal except for cervical muscle spasm and tenderness. Preoperative laboratory examination was within normal range. After the failure of standard conservative treatment, anterior 3-level ACDF was recommended.

Standard microscopic anterior ACDF approach in general anesthesia with endotracheal tube was carried out. During surgery, the patient was in supine position with the extension of the neck by the support of the shoulders with a pillow, plaster traction of both shoulders, and Fixation of the head with plaster. For the interbody spaces, PEEK cages of size 5.0 mm were used. Interpretatively there were no complications or bleeding. Surgery duration was 2 hours and 50 minutes. The patient recovery from general anesthesia was smooth with no delaying or complication. The first 18 hours after surgery were uneventful with normal postoperative cervical X-ray.

**Figure 2:**
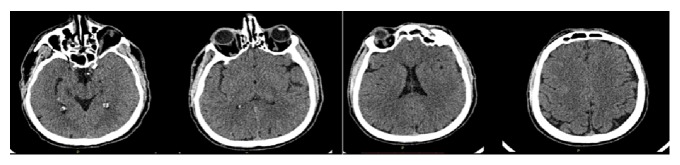
Normal finding postoperative brain CT.

However, 18 hours after surgery, he developed a sudden loss of consciousness. Clinical examination showed 8-9/15 score on Glasgow Coma Score (GCS), blood pressure 110/70 mmHg, temperature 37 degrees, pulse 84 beats per minute, oxygen saturation 98%, and blood glucose 113 mg/dl by glucometer with normal ECG. Urgent brain and cervical CT was done (Figure 2); no abnormality was detected in brain and cervical spine.

CT Angiography and MRI were not available at this time. Bilateral carotid Duplex ultrasound revealed normal blood flow in both carotid arteries. Full laboratory investigations were done and were within normal. Provisional diagnosis as stroke was done and the patient was transported emergently to the intensive care unit (ICU) for observation under heparin 1000 IU infusion/hour.

In the second postoperative day, the patient showed some improvement in level of consciousness with slurred speech, hypersomnia and loss of interest, right sided ptosis with vertical gaze palsy and diminished light reflex, memory impairment (amnesia), and right upper limb paralysis.

**Figure 3:**
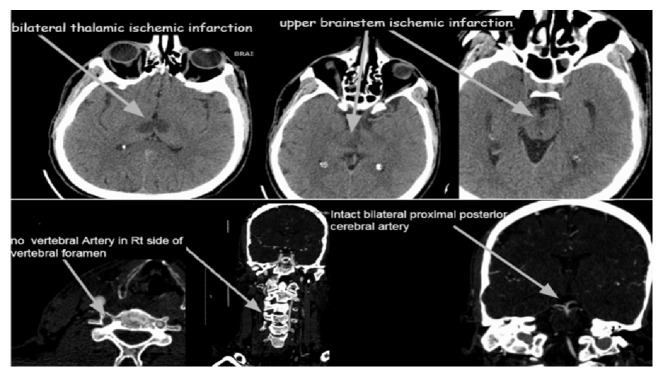
Brain and cervical CT Angiography (3rd day Post Op) revealed bilateral thalamic with upper brainstem ischemic infarction, with total occlusion of right vertebral artery from its origin and patent bilateral carotid artery with a normal flow.

On the 3rd day, CT brain and angiography revealed a bilateral thalamic with upper brainstem ischemic infarction (Figure 3), with total occlusion of Rt vertebral artery from its origin and normal flow in both carotid arteries. On the same day, the patient shifted back to neurosurgery department. Heparin infusion was stopped and shifted to clexane 80mg SC bid with the addition of cerebrolysin, ginkgo biloba+Mg, Trental, and a multivitamin.

On the 7th day after operation, patient was shifted to rehabilitation, with the improvement of the upper arm paralysis and cerebellar ataxia.

**Figure 4:**
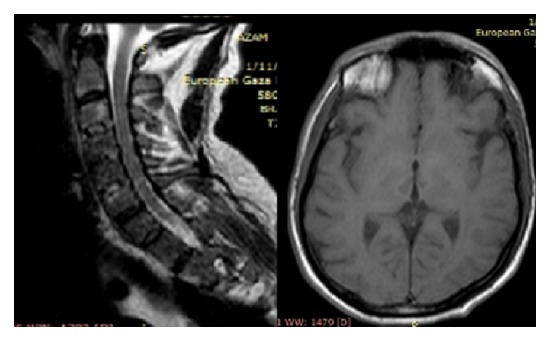
Brain and cervical MRI (after 2 weeks) show bilateral thalamic infarction with no compression on the spinal cord.

After 2 weeks, brain and cervical MRI showed a bilateral thalamic infarction with no compression on the spinal cord (Figure 4). Clinical examination after two weeks showed GCS 15/15 with normal speech, no cerebellar ataxia, no upper limb paralysis, but continuing to have hypersomnia and loss of interest with aggressive response (behavior changes), amnesia (mainly short memory amnesia), and downward gaze palsy.

**Figure 5:**
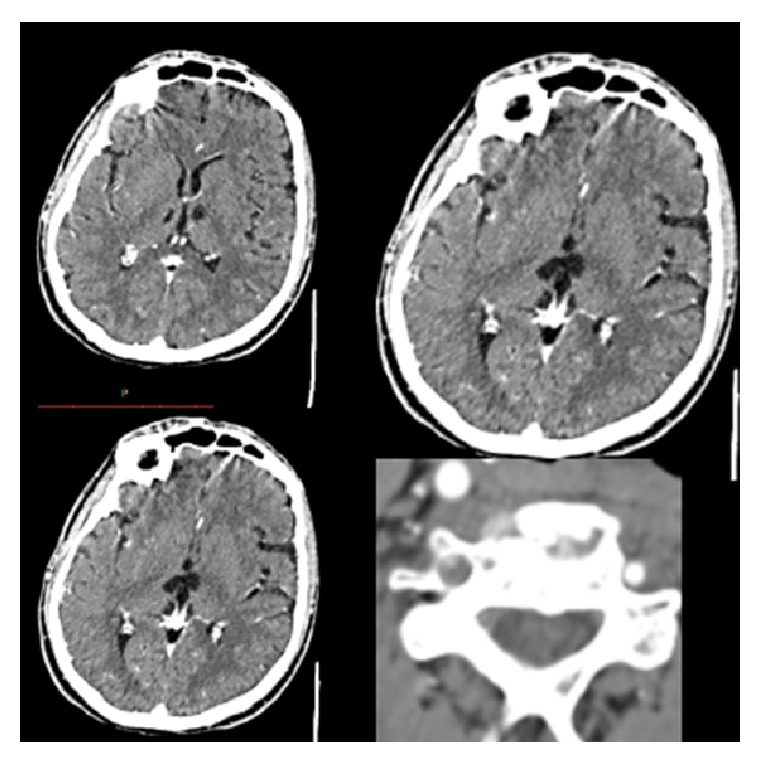
Brain and cervical CT Angiography (after 3 months) revealed a bilateral hypodense area in the site of both thalami with no recanalization of right vertebral artery.

At 3 months of follow-up, CT Angiography revealed a bilateral hypodense area in both thalami with no recanalization of right vertebral artery (Figure 5). The patient was conscious and oriented, full motor power, normal gait, no speech, sleep, and behavior deficiency. Intermittent short memory amnesia and downward gaze palsy were still present.

## 3. Discussion

ACDF is a relatively safe procedure, with ischemic stroke following Anterior Cervical Discectomy and Fusion (ACDF) being a very rare complication [2, 10]. Higher risk of postoperative stroke was reported in patients with carotid artery stenosis [5].

Stroke due to one vertebral artery occlusion has also been reported. Posterior circulation stroke and AOP occlusion are a serious complication with a higher risk of mortality and morbidity [6, 9, 11]. Yunoki et al., 2017, reported a case of right cerebellar infarction in a 50-year-old woman due to vertebral artery occlusion after ACDF [6].

A bilateral thalamic stroke is an unusual event; it raises the suspicion of the presence of the anatomical variation with the artery of Percheron, because both sides of the thalamus are supplied by the same blood supply, despite the fact that bilateral infarcts due to osculation of artery Percheron has not been established [7].

Several pathological processes may cause occlusion of VA leading to stroke. The common predisposing factor is atherosclerotic disease, dissection, fibrous banding in the neck, extrinsic compression in its second and third parts due to trauma of the cervical vertebrae or osteophyte impingement and compression, vasculitis, and arthritis [12]. Extracranial vertebral artery stenosis of the first part may account for up to 20% of posterior circulation ischemic strokes. Left side vertebral stenosis occurs more frequently [12]. Choi et al. reported a case of cerebellar infarction in 38-year-old man originating from vertebral artery stenosis caused by a hypertrophied uncovertebral joint after ACDF [13]. VA occlusion after ACDF [6], cervical fracture [14], chiropractic manipulation [15], or even after abrupt head movement [15] has been reported.

A potential mechanism of VA occlusion in such cases is intimal disruption followed by thrombus formation and, thus, clots occlusion of the vessel lumen. The reason why VA occlusion occurred, in this case, is unknown; however, repeated use of a spreader with forced distortion and/or stretching in three consecutive intervertebral spaces may have triggered this sequence of events [6, 16].

In the case of AOP, an embolism is the most frequent cause of occlusion [9]. In our case, there was vertebral artery occlusion on Rt side thrombosis from the origin. Embolus from vertebral artery to the AOP is most probably the case of the occlusion of AOP.

Interpretation of the case is challenging due to the limited evidence regarding bilateral thalamic infarction because of AOP. The scenario in our case as we assume is hyperextension injuries of the vertebral artery during the surgery. The hyperextension injury leads to disruption of intima and gradual development of thrombus. Hours after trauma, an embolism to AOP was the cause of symptoms. Our assumption is supported with published data which showed that hyperextension injuries are one mechanism of closed injury to the vertebral artery and that artery is at risk, especially in the mid-cervical region and that the fibro-osseous tunnel does not offer adequate protection [17].

López-Serna et al. reported a similar case of bilateral thalamic stroke due to occlusion of the artery of Percheron in a patient with patent foramen ovale; the outcome was similar to the outcome in our case [7].

Clinical outcome is not poor, but the initial recovery phase is crucial. If the patient does not improve in the initial phase, the chance for further recovery appears to be low; hence, in the appropriate patient, thrombolytic therapy should be considered [9].

The published cases show that the state of consciousness may spontaneously resolve during the first 3 weeks [7] with some patients without persistent neurological symptoms [6]. The outcome in our case is in agreement with published data which showed that unilateral vertebral artery occlusion seldom results in a permanent neurological deficit. However, transient neurological symptoms are not uncommon [17]. Published data showed that memory deficits, vertical gaze paresis, and hypersomnia considerably subsided in the majority of patients [18].

Our experience demonstrates the need for surgeons performing ADCF procedures to be aware of this potential complication. There are many other prophylactic treatments of this complication such as maintaining appropriate cervical positioning during surgery or prevention of postoperative dehydration; however, care should be taken to avoid intimal disruption of the VA by overly dilating the intervertebral space.
